# Experimental Investigation of the Oxidative Coupling of Methane in a Porous Membrane Reactor: Relevance of Back-Permeation

**DOI:** 10.3390/membranes10070152

**Published:** 2020-07-14

**Authors:** Aitor Cruellas, Wout Ververs, Martin van Sint Annaland, Fausto Gallucci

**Affiliations:** Department of Chemical Engineering and Chemistry, Eindhoven University of Technology, P.O. Box 513, 5600 MB Eindhoven, The Netherlands; A.Cruellas.Labella@tue.nl (A.C.); w.j.r.ververs@tue.nl (W.V.); m.v.sintannaland@tue.nl (M.v.S.A.)

**Keywords:** oxidative coupling of methane, membrane reactors, porous membrane, distributive feeding

## Abstract

Novel reactor configurations for the oxidative coupling of methane (OCM), and in particular membrane reactors, contribute toward reaching the yield required to make the process competitive at the industrial scale. Therefore, in this work, the conventional OCM packed bed reactor using a Mn-Na_2_WO_4_/SiO_2_ catalyst was experimentally compared with a membrane reactor, in which a symmetric MgO porous membrane was integrated. The beneficial effects of distributive feeding of oxygen along the membrane, which is the main advantage of the porous membrane reactor, were demonstrated, although no significant differences in terms of performance were observed because of the adverse effects of back-permeation prevailing in the experiments. A sensitivity analysis carried out on the effective diffusion coefficient also indicated the necessity of properly tuning the membrane properties to achieve the expected promising results, highlighting how this tuning could be addressed.

## 1. Introduction

The continuous trend of oil price increases, together with the growing demand for light hydrocarbon worldwide, are the main causes why interest for alternative technologies to produce light hydrocarbons is exponentially rising.

The production of ethylene can be used as a representative example of this trend. This bulk chemical is used as the precursor of many plastics. Its huge scale production, surpassing 140 million tons per year, makes its production one of the most relevant in the chemical industry. The main process employed nowadays for the production of ethylene (in Europe) is naphtha steam cracking, where oil is used as a primary feedstock. However, the already mentioned increasing oil prices, together with CO_2_ emissions derived from the naphtha steam cracking process (around 2.6 tons of CO_2_ per ton of ethylene produced [1]), makes research into alternative technologies more appealing.

A feasible alternative is to substitute oil by natural gas as feedstock, both because of its lower CO_2_ emissions and because of its price, which is increasing at a lower rate than other fossil fuels [2].

The production of ethylene from natural gas (a mixture containing mostly methane), can be accomplished via the direct route, termed oxidative coupling of methane (OCM), and the indirect route, which involves syngas production and Fischer–Tropsch synthesis. In the indirect route, natural gas is firstly converted into syngas (a mixture of CO and H_2_), while in a secondary step, higher hydrocarbons are produced and further cracked to ethylene. This route is very inefficient due to the large methane formation in the cracking step. On the other hand, the direct route aims to produce ethylene from methane in a single step through the following reaction:

(1) $$2\;CH_{4}+O_{2}\to C_{2}H_{4}+2\;H_{2}O$$

This main reaction is accompanied by many homogeneous and heterogeneous reactions in the primary and secondary steps [3]. Three primary reactions are considered to exist:

(2) $$2\;CH_{4}+\frac{1}{2}O_{2}\to C_{2}H_{6}+H_{2}O$$

(3) $$CH_{4}+2O_{2}\to CO_{2}+2H_{2}O$$

(4) $$CH_{4}+O_{2}\to CO+H_{2}$$

The complete and incomplete combustion reactions of methane, which compete with the desired formation of ethane in the primary step, significantly decrease selectivity toward the main desired reaction product. As soon as ethane starts to be produced, its oxidative and/or nonoxidative dehydrogenation for the production of ethylene can take place. However, in parallel with these reactions, the undesired consecutive reactions (combustions) also arise, reducing the final ethylene yields. Additionally, the heat released due to the mildly exothermic nature of OCM can also be an issue, since it can easily lead to hotspots if not properly controlled, which can deactivate the catalyst and provoke a steep drop in selectivity toward C_2_s.

These are the main reasons why [4] great efforts have been carried out to improve performance since the 1980s, when this process started to attract interest. In a recent study, OCM demonstrated potential to be a feasible alternative to conventional ethylene production technologies [5]. However, the authors also mentioned that, with the current state-of-the-art (which considers a C_2_ yield of around 15% at conditions somehow applicable at the industrial scale), OCM is not economically viable.

The selection of a more appropriate catalyst could contribute to maintain higher selectivity toward the desired products. The primary function of OCM catalysts is to break C-H bonds for the formation of methyl radicals, since these radicals are susceptible to the coupling reaction toward ethane. Although this mechanism is not 100% clear [6], the Mars–Van Krevelen mechanism is the most accepted hypothesis, whereby CH_4_ reacts with the O_2_ adsorbed at the active site of the catalyst to form methyl radicals, which then combine to form C_2_H_6_ [6,7]. Following this mechanism, a 100% selectivity toward C_2_ could be achieved. However, this is not realistic, since combustion of CH_4_ and methyl radicals should also be accounted for. The complexity of the reaction mechanism makes the adjustment of the process conditions to optimize the catalyst behaviour more difficult. Hence, advanced techniques, such as high-energy X-ray diffraction computed tomography, are required to acquire a deep understanding about the nature of the catalyst and its specific behaviour and changes when operating under OCM conditions [8,9,10].

Many different catalysts with specific modifications were tested for OCM. One of the first catalysts that was tested for OCM is Li/MgO, which was first developed by Ito et al. in 1985 [11]. From that point on, numerous studies focused on the improvement of its performance parameters, reaching a C_2_ yield of 20% [12]. However, its stability is still an unsolved issue because of the migration of Li species during the OCM reaction, severely lowering the selectivity of the catalyst over time [13]. Because of this issue, research migrated toward the La_2_O_3_ catalysts, widely reported and known to be highly active and with decent stability [14]. These catalysts are often doped to improve their performance. For instance, a modification containing Sr (Sr-La_2_O_3_ catalyst) was used by Choudhary et al. to obtain a C_2+_ yield of 17.1% [15]. Despite showing decent, but not industrially feasible, C_2_ yields, poor selectivity still holds back most La-based catalysts. In addition to the traditional OCM catalysts, Mn-Na_2_WO_4_/SiO_2_ recently became the most intensively investigated OCM catalyst due to its improved selectivity. Major topics regarding this catalyst involve a general understanding of the reaction mechanism and the optimal composition of the catalyst [6,16,17,18]. Even though elaborate studies investigated the exact reaction mechanism and active centres, there is still no agreement in the literature on what the catalytically active centre for OCM on Mn-Na_2_WO_4_/SiO_2_ exactly is [19,20]. C_2_ yields of above 20% for packed bed reactors were reported for this type of mixed oxide catalyst [21,22,23]. Besides promising yields, catalyst stability is probably the aspect that distinguishes Mn-Na_2_WO_4_/SiO_2_ from other OCM catalysts in the literature. Multiple studies and reviews stated that Mn-Na_2_WO_4_/SiO_2_ is the best and most practical out of all OCM catalysts currently available [19,23,24].

The second major topic historically researched to improve OCM reactor performance relates to the reactor configuration. Many different reactor configurations were attempted throughout OCM history [21]. It is generally well accepted that selectivity toward C_2_s is maintained as long as oxygen partial pressure is kept low. As an example, the well-known OCM kinetics developed by Stansch et al. [3] showed this behaviour, since the reaction order with respect to the oxygen of the primary desired reaction is lower than that of the undesired reaction. Based on this fact, distributed oxygen feeding along the reactor contributes to keep the local oxygen partial pressure low (providing high C_2_ selectivities), while maintaining overall decent CH_4_ conversion, hence resulting in C_2_ yield improvement [25]. Many alternative reactor configurations were experimentally attempted based on this principle, including the chemical looping concept [26,27,28,29,30], packed beds with multiple oxygen feedings [31,32], and membrane reactors [33,34,35,36,37,38,39,40,41,42]. Membrane reactors attracted great interest; besides homogeneous oxygen distribution, integration of membranes can be used to facilitate the heat management of the system, thus mitigating the issues derived from the mild exothermicity of the reaction (part of the heat released during OCM could be utilized by the gas flowing inside the membrane).

Two different types of ceramic membranes, dense and porous, can be employed in OCM membrane reactors. Dense membranes can selectively separate oxygen from a mixture and feed into the reactor side. These membranes, also called Mixed ionic-electronic conducting (MIEC) membranes, are commonly perovskite and/or fluorite materials [33]. Since they are oxygen-selective, their integration avoids the use of the very intensive air separation unit required in other cases to feed pure oxygen into the reactor. However, the low flux that these membranes can provide, the complex sealing that they require and their generally poor stability under OCM conditions limit their applications. Modifications of conventional, single-phase, self-supported membranes are currently being investigated (leading to the development of supported and/or dual-phase, oxygen-selective membranes), but all the issues aforementioned are as yet unsolved.

Porous membranes are an alternative to oxygen feeding distribution throughout the axial length of the reactor. Differently from dense membranes, porous membranes are not selective, thus, the air separation unit cannot be removed from the system during their employment. On the other hand, their sealing can be more easily accomplished and their stability at high temperature was extensively proven [43]. In addition, the flux through these membranes can be tuned depending on the necessities of the experiments by changing the retentate-permeate pressure difference, hence giving more flexibility to the experimental system. If we also consider that Cruellas et al. [44] recently showed that the integration of porous membranes makes ethylene cheaper than when dense membranes are employed in an industrial-scale OCM plant, the selection of porous membranes was warranted to carry out the experimental part of this work.

Even though large research efforts are being made regarding both types of membranes and their suitability to be integrated into membrane reactors, detailed experimental comparisons between membrane reactors and conventional reactors are lacking in the literature.

Therefore, the main objective of this work is the experimental comparison between packed bed and packed bed membrane reactor performances, highlighting the main parameters that should be optimized to maximize the theoretical OCM performance improvement expected with the membrane reactor. 

## 2. Materials and Methods

### 2.1. Catalyst Preparation

The Mn-Na_2_WO_4_/SiO_2_ catalyst used in this work was prepared and provided by Johnson Matthey by incipient wetness impregnation. The final formulation of the catalyst was 1.6%Mn-5%Na_2_WO_4_. The catalyst was crushed and sieved to obtain the desired particle size, between 250 and 355 µm. The BET surface area of the catalyst was measured with a ThermoFischer Surfer (Milano, Italy) and found to be 17.01 m^2^/g, while its skeleton particle density was 1693 kg/m^3^. For all the experiments carried out, the catalyst was diluted by 50% with quartz of the same particle size. In this way, the amount of heat released was decreased, making the temperature in the bed more homogeneous and simplifying the heat management issue.

### 2.2. Membrane Preparation

**Figure 1:**
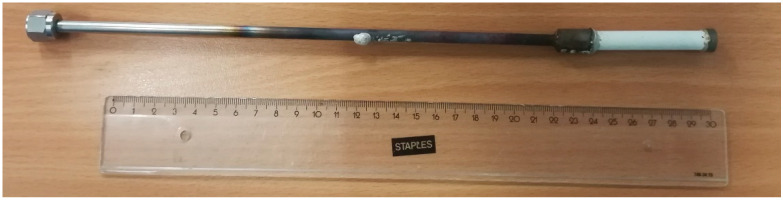
Picture of the sealed (by means of the reactive air brazing (RAB) sealing technique) MgO porous membrane used to carry out the membrane reactor experiments.

As stated in the introduction, porous membranes were used to build up the OCM membrane reactor because of their simplicity and comparable performance (in terms of economic results when integrated in a membrane reactor belonging to an industrial-scale OCM plant) to dense membranes. In particular, symmetric MgO tubes prepared by Rauschert wer used as porous membranes. These membranes were sealed to FeCr-alloy reducers and caps using the reactive air brazing (RAB) sealing technique [45] and then welded to dense metallic tubes for integration into the reactor. A picture of the sealed membrane can be seen in Figure 1.

### 2.3. Experimental Procedure

**Figure 2:**
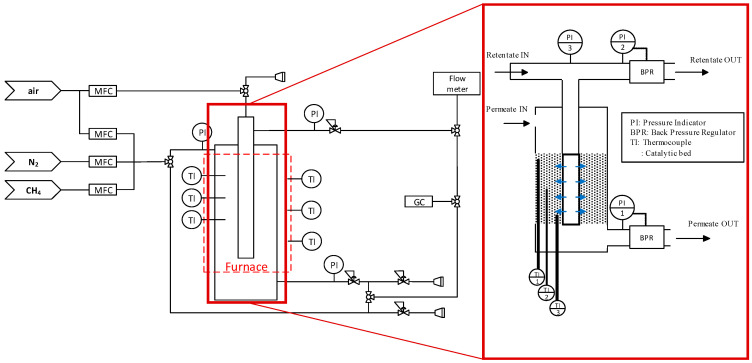
Scheme of the setup used for the experiments both with packed bed and packed bed membrane reactor configurations, with zoom-in to the reactor part.

The setup used to carry out the experiments was composed by different sections, namely, a feeding section, a reactor section, and an analysis section. In general, four streams were considered for this system, that is, permeate inlet, permeate outlet, retentate inlet, and retentate outlet. The scheme of the setup is depicted in Figure 2.

By means of a set of valves, all the streams could be independently sent to the analysis section to determine their composition (in a micro-GC) and/or to the flowmeter to quantify the volumetric flow rate. This strategy allowed for the calculation of the carbon balance in each of the experiments carried out. Carbon atom mass balances of the results reported herein were within 2%, and in most cases even within 1%; thus, the results were considered to be sufficiently accurate for analysis and discussion.

#### 2.3.1. Reactor Section

**Figure 3:**
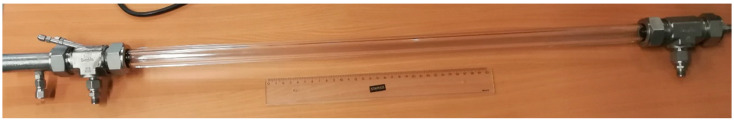
Picture of the empty reactor used to carry out the experiments.

The reactor consisted of a quartz tube, expected to be inert and sufficiently resistant to the temperatures required for OCM, sealed by means of graphite to two metal connections. A picture of the empty reactor is shown in Figure 3. 

**Figure 4:**
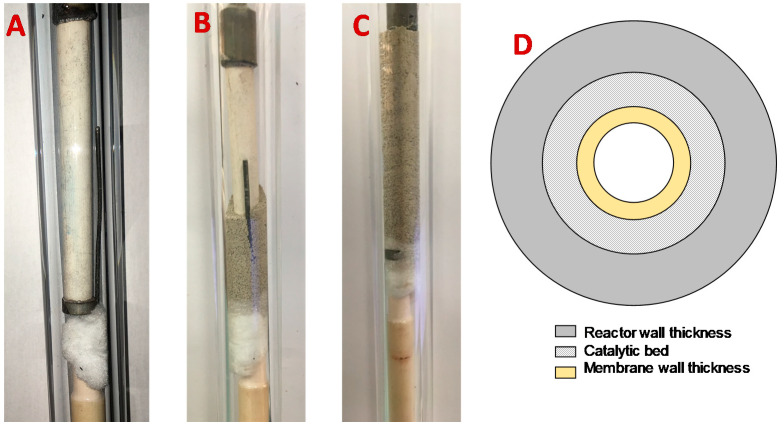
Pictures of the membrane reactor without catalyst (**A**), when part of the catalyst was placed (**B**), and when the catalytic bed was completed (**C**), and the scheme of the view of the reactor in cross-section (**D**).

The reactor, with a length of 70 cm, was longer than required to make sure that both sealings were cold and gas tight. Three thermocouples were introduced in the reactor bed at different axial positions to monitor the temperature along the reactor, while another thermocouple was placed in the retentate side (inside of the membrane). The pressures on both sides were controlled by means of back-pressure regulators, while the pressure indicators for the different reactor sections were used to control the pressure drops in these regions. In addition, the back-pressure regulator of the retentate side was used to adjust the retentate–permeate pressure difference. This transmembrane pressure difference was the driving force that governed the system and determined the amount of gas permeating via the membrane from the retentate to permeate side. The membrane was firstly sealed independently to a metallic tube, then this metallic tube was connected to the reactor via its correspondent Swagelok connection. Subsequently, the catalyst was placed inside the reactor, where glass wool was used to hold the catalytic bed, as shown in Figure 4.

In addition, bigger quartz particles were placed on top of the bed from where the inlet gas originated, in order to pre-heat the gas up to the desired temperature. Finally, the reactor was located inside a furnace for temperature control.

#### 2.3.2. Analysis Section

After the reactor, the retentate and permeate outlet gases were chilled and liquids were condensed in two condensers. The feed and dry product streams were analyzed by online gas chromatography (GC), with a Varian Micro-GC 4900 containing three columns, two 5°A molsieve columns to separate oxygen, nitrogen, methane, carbon monoxide, and hydrogen, and a PoraPLOT Q column to separate H_2_O, CO_2_, C_2_, and C_3_ components. The columns were equipped with thermal conductivity detectors. Helium was used as the internal standard.

### 2.4. Reactor Configuartions

A summary of the conditions that all the experiments had in common is given in Table 1.

**Figure 5:**
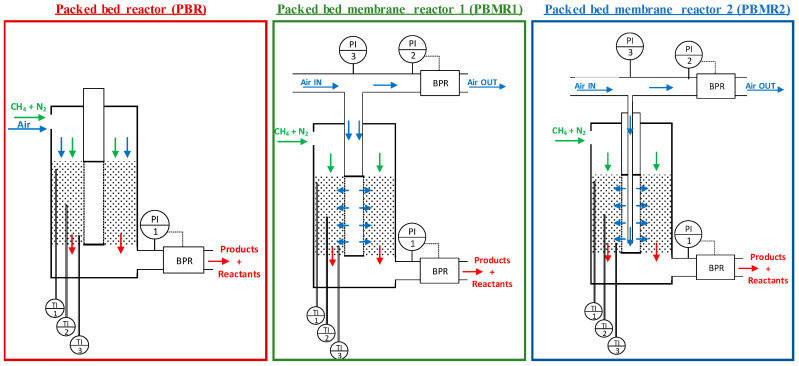
Scheme of the different reactor configurations (packed bed reactor (PBR), packed bed membrane reactor 1 (PBMR), and packed bed membrane reactor 2 (PBMR2)) used for this study.

The temperature was controlled such that none of the three thermocouples placed inside the bed exceeded 800 °C. The reactants, methane, and, in case of the conventional packed bed reactor, air were always 70% mixed with N_2_ to dilute the reaction and minimize the effect that the exothermicity of the OCM reaction could cause on the axial temperature profile in the reactor. To assure permeation from the retentate to the permeate, the retentate pressure was always kept higher than the permeate pressure. As explained later, the pressure in the retentate was controlled to determine the amount of air permeating into the reaction side, which was maintained during all the experiments at 2 bar. Flow rates were adjusted to provide reasonable residence times of the gases into the reactor, while the CH_4_/O_2_ ratio varied, as this was one of the most important parameters analyzed in this work. The dimensions of both the membrane and the reactor were selected in order to limit radial concentration profiles when the membrane reactor configuration was used. Finally, the thick wall of the quartz reactor was chosen to avoid problems when increasing the pressure and to increase the mechanical strength of the reactor. With all these conditions, the aim of this work was to compare the conventional (co-fed) packed bed reactor with the packed bed membrane reactor. To do so, three different reactor configurations were employed, which are detailed in Figure 5.

For the packed bed reactor (PBR) configuration case, the so-called “retentate side” remained closed (no gas was fed from there), and all the reactants were co-fed from the “permeate” side. Subsequently, the gases went through the whole catalyst bed.

In the packed bed membrane reactor 1 (PBMR1), methane and gas diluent (N_2_) were fed from the permeate side, while air was fed to the retentate side. The back-pressure regulator (BPR) located at the retentate outlet allowed control of the amount of air permeating to the permeate side. Thus, the total air fed was distributed by the air flow always fed in excess to allow the PBR to set the desired pressure in the retentate, whereby part of it reached the membrane and permeated (the amount depending on the retentate–permeate pressure difference), while the rest went through the BPR and leave the system. Contrary to many other experiments, in which all the flow was aimed to permeate through the membrane, in this system, the characteristics of the membrane together with the ΔP were the parameters governing the permeation. Differently to PBMR1, where the air flowed freely inside the membrane, a thin tube was introduced into the retentate side of the packed bed membrane reactor 2 (PBMR2) to direct the air flow until the end of the membrane. At that point, the air reached the retentate side and started to permeate. The reason behind carrying out experiments with the PBMR1 and PBMR2 configurations was to determine whether the axial position in which air was fed into the retentate side (at the beginning or at the end of the membrane) affected the results.

## 3. Results

### 3.1. Mass and Heat Transfer Limitations

The relevance of possible heat and mass transfer limitations in a catalytic bed were quantified to verify that the observed reaction rates matched with the reaction rates predicted from the determined reaction kinetics. 

The general procedure for determining mass and heat transfer criteria is explained in Appendix A.

In the calculations regarding mass and heat transfer, oxygen was used as the potentially limiting reactant and thus the most important species. The mass and heat transfer criteria were checked for a standard case of OCM in a conventional packed bed reactor. Conditions of this “standard” case are shown in Table 2.

The calculated mass and heat transfer criteria are shown in Table 3. Each criterion was determined for two different situations. Cases with different assumptions were indicated with letters a–f, and the intraparticle effectiveness factor was also determined. Detailed calculations of mass transfer can be found in Appendix A.

The Mears criterion for external mass transfer was determined using the Sherwood correlation given in Appendix A. However, the calculated Reynolds number of 1.51 exceeded the provided accuracy limits of the Sherwood correlation [46]. To put this in perspective, a worst-case scenario was also supplied to the Mears criterion. This worst-case scenario (case b) was the case where external mass transfer was fully diffusional, leading to a Sherwood number of 2. The results of the Mears criterion calculation showed that even in the worst-case scenario (case b), external diffusion limitations were absent.

Secondly, the presence of internal mass transfer limitations was checked using the Weisz–Prater criterion. Case c calculated the effective diffusivity for catalyst pore size (6.5 nm) in the mesoscale regime, since this was reported for a similar type of catalyst, while case d used a catalyst pore size (50 nm) in the macroscale regime [47]. For both case c and d, it was found that internal mass transfer limitations could be neglected. Besides the Weisz–Prater criterion, the effectiveness factors were also calculated for both cases, obtaining values of 97.4% and 99.4%, confirming that internal mass transfer limitations hardly influenced the results.

The abovementioned results corresponded to what Tiemersma et al. [48] showed using both calculations and experiments for a similar system.

The heat transfer criteria were also verified for two different cases; these results are summarized in Table 3.

### 3.2. Packed Bed versus Packed Bed Porous Membrane Reactor

The performance of the conventional packed bed configuration was compared to the packed bed membrane reactor with the integrated MgO porous tube.

#### Membrane Characterization

First, experiments without reaction were carried out to quantify the membrane permeation rate as a function of the transmembrane (permeate–retentate) pressure difference. The conditions of these experiments are summarized in Table 4.

To measure the membrane flow (which is then converted to membrane flux) at certain conditions, the outlet of the retentate also needed to be measured. This value was subtracted from the retentate inlet flow rate to obtain the permeation value. This procedure was repeated for all of the cases.

**Figure 6:**
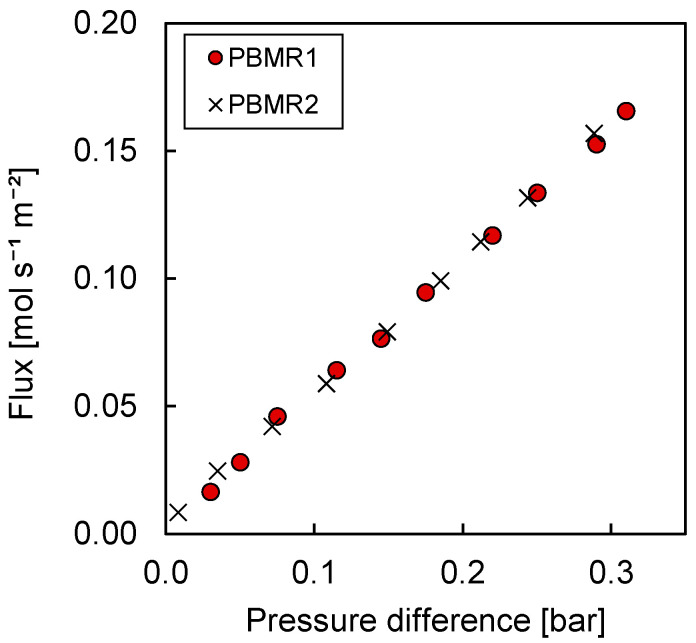
Flux of the symmetric MgO porous membrane plotted against the retentate–permeate pressure difference.

The membrane flux was measured both with PBMR1 and PBMR2 configurations. The results are shown in Figure 6.

The results showed that the way in which air was fed to the retentate side did not influence the membrane flux that was achieved when no reactions took place. Thus, in absence of any reaction, the pressure was constant along the axial coordinates of the retentate side (otherwise differences between the PBMR1 and PBMR2 configurations would have been observed). Moreover, the pressure drop caused by the thin tube introduced in PBMR2 was shown to be negligible. The results of these tests were generally applicable for the design of the OCM reaction tests discussed in the next section, where the transmembrane pressure difference required to obtain the desired CH_4_/O_2_ ratios for the OCM reaction experiments was calculated based on the information acquired in this section.

Prior to these experiments, other membrane tests (without reaction) were also performed. Permeation was measured at different temperatures and for several gases, with these tests reported in Appendix B. The flux variability observed when employing different gases and the higher fluxes obtained when feeding a light gas (He) supported the strong influence of Knudsen diffusion in the permeation mechanism of the porous membrane of study. The presence of Knudsen diffusion is relevant to this work and is discussed in the upcoming sections (namely, Section 3.3.1) to explain and justify some of the results achieved when carrying out the OCM reaction. In particular, this Knudsen diffusion mechanism implies the back-permeation of some species during the OCM membrane reactor experiments.

### 3.3. Membrane Reactor Experiments

**Figure 7:**
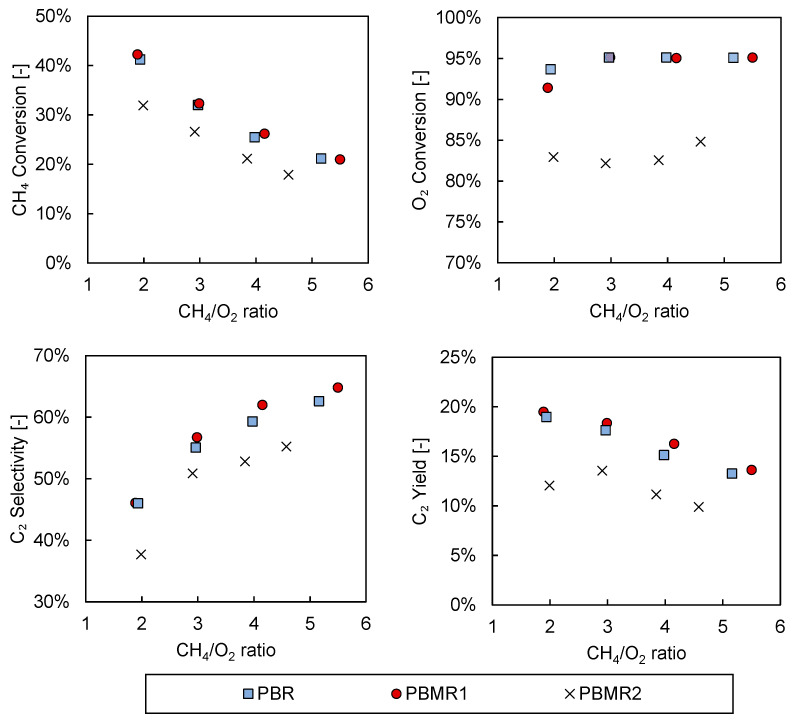
CH_4_ conversion (top left), O_2_ conversion (top right), C_2_ selectivity (bottom left), and C_2_ yield (bottom right) of the different reactor configurations (PBR, PBMR1, and PBMR2) employed for oxidative coupling of methane (OCM) in experiments carried out at 800 °C, 2 bar, and with a CH_4_ inlet flow of 105 mL/min.

To investigate the effect of the integration of a membrane into the reactor on the performance of the OCM process, experiments with the conventional packed bed were first carried out, which were then compared with both membrane reactor configurations (PBMR1 and PBMR2). The results of this comparison on the basis of CH_4_ conversion, O_2_ conversion, C_2_ selectivity, and C_2_ yield as functions of the CH_4_/O_2_ ratio are summarized in Figure 7.

In general, the obtained performance followed the expected trend as a function of the CH_4_/O_2_ ratio. The selectivity toward the desired products decreased while both reactant conversions increased when operating at low CH_4_/O_2_ ratios. At lower CH_4_/O_2_ ratios, more oxygen was available to react with the methane, thereby increasing its conversion. However, the higher oxygen concentration favored undesired reactions (that are enhanced when high oxygen partial pressure is high), resulting in a decrease in the overall C_2_ selectivity. In terms of the C_2_ yield, an optimum was observed at a CH_4_/O_2_ ratio of around 2–3, depending on the reactor configuration, thereby balancing the C_2_ selectivity and CH_4_ conversion.

The obtained results were in agreement with the literature [25,49], indicating that both the catalyst was active at the conditions of the experiments and that the operating conditions employed in this study were reasonable and led to consistent results. Specifically, the 20% C_2_ yield reached using a packed bed reactor was on the higher edge of the published experimental work, and was already better than many other experimental works reported in the literature [21]. In addition, this work was carried out with a relatively large packed bed, i.e., using reaction beds longer than 5 cm and with an amount of catalyst exceeding 5 grams in most of the cases.

**Figure 8:**
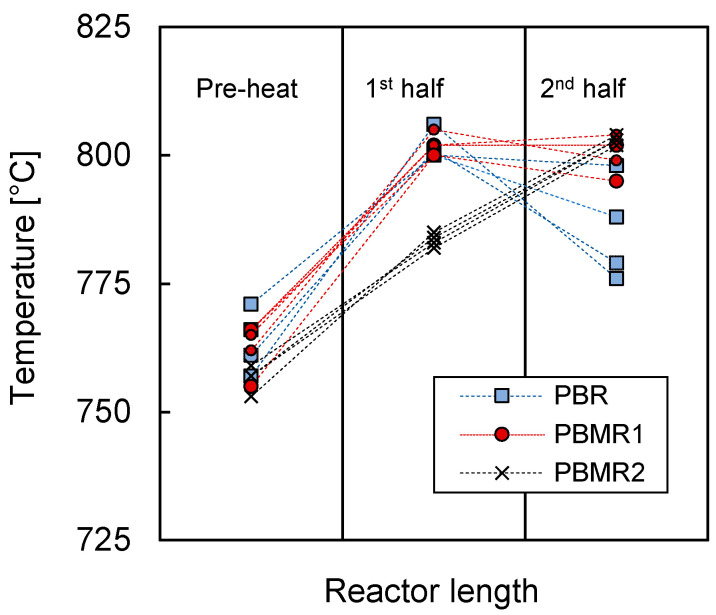
Temperature distribution in the reaction bed for all the experiments carried out in this work, divided according to PBR, PBMR1, and PBMR2.

The difference in the results of both membrane reactor experiments (PBMR1 vs. PBMR2) was not expected, however. as the measured membrane flux was the same for the two configurations. These results highlighted the fact that the way in which air was fed into the retentate side of the system did have a significant influence on the OCM performance. The lower conversion of the reactants in the PBMR2 configuration could indicate that, in this case, the permeation of air was not uniform and was higher toward the end of the catalytic bed, where the O_2_ had a much reduced residence time to react with the available CH_4_, thereby decreasing both the CH_4_ and O_2_ conversion. This nonuniform air permeation could also have influenced the thermal behavior of the reactor, since in these conditions most of the heat would be generated at the end of the bed. The difference in this thermal behavior could well be the origin of the lower C_2_ selectivity achieved with this configuration. To study this effect in more detail, the temperatures were monitored during all the experiments; these are shown in Figure 8:

The axial temperature profile in the PBMR2 showed that the bed was heated most toward the end of the bed, most likely because of the heat released during the exothermic OCM reaction in that specific region of the reactor. The reason for this is that in PBMR2 most of the oxygen was fed in the second half of the reactor because of the oxygen feeding policy previously described. On the other side, PBMR1 showed the highest temperature point in the first half of the bed, where most of the oxygen was expected to permeate. In addition, the temperature profile of the PBMR1 notably differed to that of PBR. The temperature profile of PBMR1 was more flattened, indicating that the reaction and consequently the heat was distributed more evenly along the bed. On the contrary, the temperature profiles of the PBR were steeper, and most of the reaction occurred at the very beginning of the bed due to the co-feeding of methane and oxygen, which was justified by the fact that toward the end of the bed, the temperature in the PBR dropped much more than in the case of PBMR1. Therefore, the differences in these temperature profiles corroborated the idea of more even oxygen distribution throughout PBMR1 and PBMR2.

However, the differences in temperature profiles, and thus the oxygen distribution along the bed, did not seem to correspond to the results shown in Figure 7, where there was no significant difference observed between PBR and PBMR1. A more homogeneous oxygen distribution (PBMR1) should have led to higher C_2_ selectivities, especially at lower CH_4_/O_2_ ratios. This indicated that the permeation rate was not uniform along the reactor, which limited the maximum performance that could be achieved with PBMR1. The nonuniformity of the permeation flux along the reactor is investigated in more detail in the next section.

#### 3.3.1. Permeation Mechanisms through the Porous Membrane

From the temperature profiles discussed so far, it is clear that when employing the porous membrane reactor, the permeation of oxygen was not homogeneous along the reactor length, despite the fact that it was demonstrated that the pressure was constant along the retentate side of the system. The pressure along the retentate side could be measured because of the thin tube placed there; indeed, a uniform permeation profile was obtained (see Figure 6) when no reactions took place, independently from where the oxygen was fed. The nonuniformity of the membrane permeation during the reactive experiments and the lack of differences between the PBR and PBMR were related to the different permeation mechanisms that can occur through a porous membrane. The equation governing the permeation flux through a porous membrane is given by

(5) $$J_{i}=\frac{1}{\mathrm{RT}}\left(D_{\mathrm{eff}}\frac{\partial \left(x_{i}P\right)}{\partial r}+\frac{B_{0}x_{i}P}{\mu }\frac{\partial P}{\partial r}\right)$$

where J_i_ (mol∙s^−1^∙m^−2^) is the permeation flux of component i, D_eff_ (m^2^∙s^−1^) is the effective diffusion coefficient of the species i in the membrane, and B_0_ (m^2^) the convective permeability constant. D_eff_ was assumed to be 10^−6^ m^2^∙s^−1^ and B_0_ was fitted using the measured permeation data and was determined to be 2 × 10^−14^ m^2^. The effective diffusion coefficient depends on the membrane morphology and combines Knudsen and molecular diffusion into one lumped coefficient, D_eff_. P refers to the pressure, T is the temperature, R is the universal gas constant, x_i_ is the fraction of component i, μ (Pa∙s) is the dynamic viscosity, and r refers to the radial direction.

In work by Aseem et al., it was stated that “In the absence of a sufficient transmembrane pressure gradient, back-diffusion of tube side reactant to the feed side can occur” [50]. In addition, it was also shown that the morphology of the membrane should be tailored in order to minimize back-diffusion [51]. Because the membrane used in this work showed relatively high permeance, the permeate–retentate pressure difference needed to reach the desired flux was generally small, and therefore it indeed allowed for some back-diffusion to occur.

**Figure 9:**
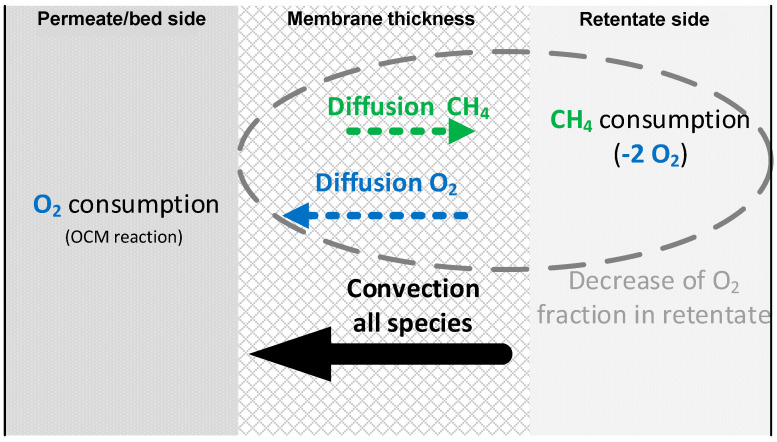
Schematic summary of the effect of diffusion and convection on membrane permeation.

In the system of study, there is firstly a convective term with the direction of retentate to permeate, a diffusive O_2_ flux going to the permeate side and a diffusive flux of methane (also products) back-permeating into the membrane. This back-permeation of CH_4_ not only removes one of the OCM reactants from the catalytic bed, but also burns part of the oxygen intended to be fed to the reaction side (the retentate side still exists at a high temperature and combustion occurs if CH_4_ and O_2_ encounter each other). Actually, two O_2_ molecules are needed to burn (combust) a single CH_4_ molecule. On top of this, as soon as the OCM reaction takes place in the permeate side, C_2_ and CO_x_ start to be formed. These molecules could also, at a certain point, back-permeate into the retentate side, where they could be oxidized further by the oxygen still present there. Therefore, it can be said that the combustion of back-permeated species amplifies the effect of back-diffusion on the oxygen distribution. If the fraction of oxygen in the retentate decreases because of combustion taking place, the oxygen content of the convective permeation term also decrease, leading to a nonuniform oxygen permeation profile. All these phenomena are schematically represented in Figure 9.

Based on the extended Fick’s law equation and all the extra phenomena taking place in the system of study, a 1D model with reactor–membrane exchange of species was built to study the evolution of the species at both the retentate and permeate sides, attempting to explain the experimentally observed behaviour. The model was a plug flow of basic mass balances, using a 10-reaction OCM kinetic model for a similar catalyst [52].

Steady state mass balances for the 1D model are given below.

(6) $$\frac{{\mathrm{dF}}_{i}}{\mathrm{dz}}=R_{i}A+J_{i}\;2{\pi R}_{\mathrm{mem}}$$

where $F_{i}$ is the molar flow rate of component i, $R_{i}$ is the reaction rate of component i per unit volume of reactor, and $J_{i}$ is the molar permeation flux. A is the cross-sectional area of the reactor/bed and z represents the axial direction.

The reaction rate is determined by kinetics described by Danespayeh et al. [52] corresponding to the Mn/Na_2_WO_4_/SiO_2_ catalyst. To calculate the rate of the disappearance/production of certain components, the following expression can be used.

(7) $$R_{i}=\frac{w_{\mathrm{cat}}m_{\mathrm{bed}}}{V_{R}}\overset{10}{\underset{j=1}{\sum }}\nu_{i,j}r_{j}$$

where $r_{j}$ is the calculated reaction rate of reaction j per unit mass of catalyst and $\nu_{i,j}$ is the stoichiometric coefficient of component i in reaction j.

The flux through the membrane was described using the so-called extended Fick’s law, which is shown and explained in Equation (5).

The bed pressure drop was accounted for by including the Ergun equations, since these determined the pressure profile in the bed and, therefore, the permeation profile [53].

(8) $$\frac{\Delta p}{\Delta L}=150\frac{{\left(1-\varepsilon \right)}^{2}}{\varepsilon^{3}}\frac{{\mu u}_{s}}{{\bar{d_{p}}}^{2}}+1.75\frac{1-\varepsilon }{\varepsilon^{3}}\frac{\rho_{g}u_{s}^{2}}{\bar{d_{p}}}$$

where ε is the bed porosity, μ is the dynamic viscosity, $\rho_{g}$ is the gas density, d_p_ is the particle diameter, and $u_{s}$ is the superficial gas velocity.

**Figure 10:**
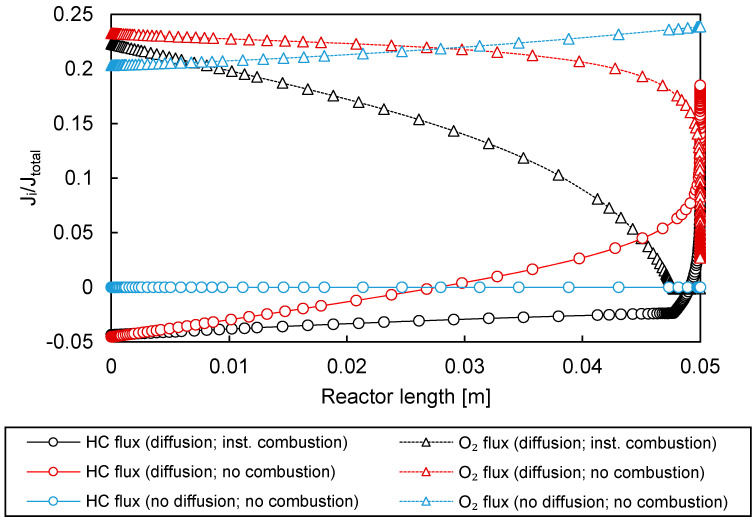
Normalized oxygen and hydrocarbon (HC) fluxes obtained from the 1D PBMR model for three cases: just convection (blue), convection and diffusion (red), and convection, diffusion, and combustion in the retentate side (red).

In Figure 10 shows how the 1D model was used to calculate the normalized hydrocarbon and oxygen fluxes, with the results plotted for three different cases. In the first case, just convection was considered, where the diffusion going through the membrane was set to zero. Subsequently, in the second case, diffusion inside the membrane was described by the extended Fick’s model, but without combustion at the retentate side. Finally, the third case included the full extended Fick’s equation and the instantaneous combustion inside the membrane (retentate side). All the fluxes were normalized by dividing the average net permeation flux ${\bar{J}}_{\mathrm{tot}}$.

The diffusive contribution clearly changed the oxygen flux profile significantly. For the case where just convection was accounted for, the only active mechanism was the convective transport of oxygen from the retentate to the permeate side. This oxygen flux increased when moving along the axial length of the reactor, because the pressure drop of the permeate side (where the catalyst was present) increased the driving force (i.e., the pressure difference between the retentate and permeate sides). In the second case, where diffusion was included, the oxygen flux decreased toward the end of the membrane as a result of a decrease in the oxygen fraction throughout the membrane. In parallel, there was also back-permeation of the OCM species, both reactants and products, from the permeate to the retentate (this permeate–retentate direction was the cause of the negative value shown in the graph). Lastly, when instantaneous combustion of hydrocarbons was included in the retentate side, the nonuniformity of the oxygen flux was even more pronounced than in the other two cases. As a result, the flux of hydrocarbons into the membrane was increased since, as opposed to the case in which combustion in the retentate was not considered, the driving force for hydrocarbon diffusion remained due to the instantaneous reaction at the retentate side. 

The observed trends in Figure 10 show that back-permeation could indeed make the oxygen distribution profiles nonuniform, corresponding with observations from the experiments. The rather small differences in performance between PBR and PBMR shown in Figure 7 and the altered behaviour for the different feeding strategies can therefore qualitatively be explained.

#### 3.3.2. Influence of Effective Diffusion Coefficient

**Figure 11:**
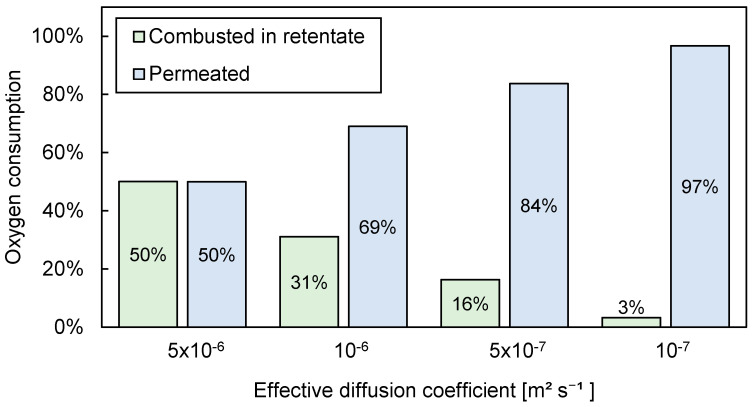
Sensitivity of effective diffusion coefficient using the distribution of oxygen consumption.

In the calculations discussed in the previous section, the contribution of diffusion to the overall flux was estimated and qualitatively described. Therefore, a sensitivity study was performed to provide a more quantitative indication regarding the diffusional effect. Four different values of the effective diffusion coefficient were tested in the 1D PBMR model with the extended Fick’s description of the permeation flux. The results of the model are shown in Figure 11, where an inlet flow rate of 370 NmL/min (70% N_2_ diluted), a bed pressure (permeate side) of 2 bar, a membrane pressure (retentate side) of 2.15 bar, and a CH_4_/O_2_ ratio of 3 were used.

It can be clearly seen that the effective diffusion coefficient plays a major role in the system. The higher the effective diffusion coefficient, the larger the contribution of oxygen consumption in the retentate. In the range of 10^−7^ to 5 × 10^−6^ m^2^·s^−1^, the consumption of oxygen via combustion in the retentate increased from 3% to 50%, indicating that the diffusion mechanism significantly hindered the maximum achievable performance in the bed, that is, part of the oxygen which was fed into the retentate side to permeate to the catalytic side (permeate) was instead consumed in the retentate side due to the diffusive back-permeation of both reactants (CH_4_) and products (C_2_H_6_, C_2_H_4_) of the OCM. Therefore, since the retentate was free of catalyst and only gas-phase combustion was considered in that compartment, the selectivity of the overall OCM process was strongly decreased. 

In summary, the fact that the C_2_ selectivity was lower than the theoretical predictions assuming a uniformly distributed feed was found to be partially caused by maldistribution of the oxygen feed over the bed, with argument that the origin of this maldistribution was the back-permeation of the hydrocarbon reactants and products. The consequence of back-permeation was that species moved from the permeate side to the retentate side, causing a nonuniform oxygen flux profile. In addition, combustion reactions caused by back-permeation were found to amplify this effect due to the consumption of oxygen in the retentate side. The nonuniformity in the oxygen flux profile caused a lower selectivity toward C_2_s and greater oxygen conversion, which was further aggravated by their influences on the temperature profile.

## 4. Conclusions

The main objective of this work was to conduct an experimental comparison of the performance of packed bed and packed bed membrane reactors for the oxidative coupling of methane (OCM) process using a Mn-Na_2_WO_4_/SiO_2_ catalyst. With the verification of theoretical calculations’ absence of internal and external mass transfer limitations, conversion in the catalyst bed was demonstrated to be governed by reaction kinetics. 

Subsequently, the OCM performance achieved with the conventional packed bed was compared with two different reactor configurations, in which a porous symmetric MgO membrane was integrated. The experiments in the packed bed reactor with co-feed of the reactants showed the suitability of the used catalyst, reaching C_2_ yields of around 20%. Even though improved results were expected with the integration of a membrane into the reactor, no significant improvement was observed in comparison with the packed bed. The differences in temperature profiles among the three employed reactor configurations indicated that the oxygen permeation flux was nonuniform along the reactor.

The cause of the absence of improvement in performance when using the membrane reactor were identified by investigating the transport mechanism of the gas through the porous membranes employed for this work. The relatively large pore size of these membranes resulted in a significant effect of diffusive back-permeation of hydrocarbon species to the retentate side, where part of the oxygen underwent combustion and should have been permeated to the catalyst bed where the OCM reaction was performed. These results highlighted the crucial importance of carefully tuning the effective diffusion coefficient, depending mainly on the morphology of the porous membrane selected, in order to avoid back-permeation and hindrance of the benefits of the membrane reactor configuration. Membranes with much smaller pore sizes or asymmetric membranes, which commonly have small layers with very small pore sizes, could help to decrease the effective diffusion coefficient, thereby minimizing the effect of back-permeation and consequently attaining higher C_2_ yields. Another solution to overcome the issues encountered in this work is the use of oxygen-selective membranes, which avoid the back-permeation effect completely. These membranes are theoretically able to transport pure oxygen to the reactor side, provided that sealing issues and leakages or pinholes are controlled. As explained in the introduction, besides the complex sealing required, the mechanical and chemical stability under OCM conditions and the low flux that they provide hinder and make their application in OCM membrane reactors difficult.

## Figures and Tables

**Table 1:** Summary of conditions and parameters used for the experiments carried out in this work.

Condition	Value
Temperature [°C]	800
Gas dilution [%]	70 (N_2_)
Catalyst dilution [%]	50 (quartz)
Type of catalyst	Mn-Na_2_WO_4_/SiO_2_
Amount of solid in the bed [g.]	3–6
Permeate pressure [bar]	2
Retentate pressure [bar]	2.1–2.3
Total flow [NmL/min]	250–750
CH_4_/O_2_ ratio	2–10
Membrane type	Porous MgO
Membrane length [mm]	50
Membrane outer diameter [mm]	10
Membrane inner diameter [mm]	7
Reactor diameter [mm]	16

**Table 2:** Conditions and performance parameters of the standard experiment used for mass and heat transfer calculations.

Condition	Value
Inlet flow rate [mL/min]	360
CH_4_/O_2_ ratio	5
Gas dilution	70% (N_2_)
Temperature [°C]	800
Pressure [bar]	2
Observed CH_4_ conversion [%]	14.5
Observed O_2_ conversion [%]	45.8

**Table 3:** Results of mass and heat transfer calculations.

Criterion	Value		Restriction
Mears criterion (external mass transfer)	0.056 ^a^	0.11 ^b^	<0.15 ^a,b^
Weisz–Prater criterion (internal mass transfer)	0.41 ^c^	0.086 ^d^	<1 ^c,d^
Effectiveness factor (intraparticle)	0.974 ^c^	0.994 ^d^	−
Mears criterion (external heat transfer)	6.38 × 10^−4 e^	7.23 × 10^−4 f^	<0.0063 ^e^ <0.0136 ^f^
Mears criterion (internal heat transfer)	1.53 × 10^−5 e^	1.74 × 10^−5 f^	<0.0315 ^e^ <0.0679 ^f^

^a^ Calculation of external mass transfer using Sherwood correlation in Appendix A; ^b^ calculation of external mass transfer using worst-case scenario of Sh=2; ^c^ calculation of internal mass transfer using mesopore size; ^d^ calculation of internal mass transfer using macropore size; ^e^ calculation of heat transfer using the methane coupling reaction; ^f^ calculation of heat transfer using the methane combustion reaction.

**Table 4:** Operating conditions of the permeation experiments.

Operating Condition	Value
Retentate inlet flow rate [NmL/min]	545
Permeate inlet flow rate [NmL/min]	545
Retentate gas composition [%]	79% N_2_, 21% O_2_ (air)
Permeate gas composition [%]	79% N_2_, 21% O_2_ (air)
Retentate pressure [bar]	From 2.01 to 2.3
Permeate pressure [bar]	2
Temperature [°C]	700
Membrane type	MgO
Membrane outer diameter [mm]	10
Membrane inner diameter [mm]	7
Membrane length [mm]	50

**Figure A1:**
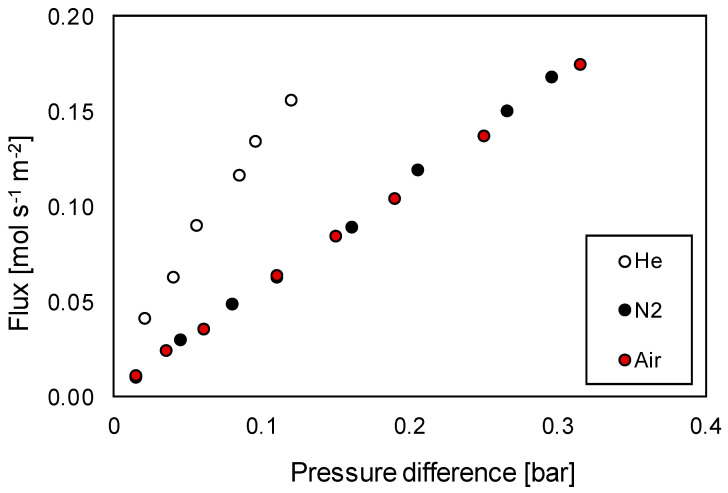
Flux of the symmetric MgO porous membrane plotted against the retentate–permeate pressure difference when different gases were fed in the retentate side. The experiments were carried out at 800 °C with a permeate pressure of 2 bar. The membrane used was the one described in Table 4.

**Figure A2:**
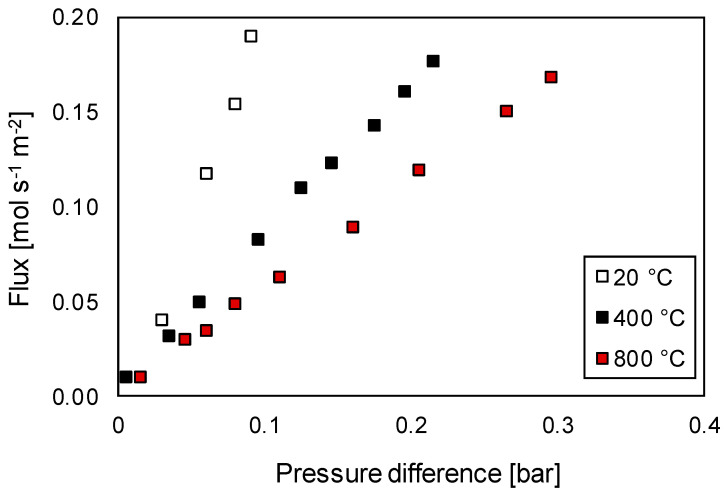
N_2_ Flux of the symmetric MgO porous membrane plotted against the retentate–permeate pressure difference at different temperatures. The experiments were carried out with a permeate pressure of 2 bar and by feeding N_2_ in the retentate side. The membrane used was the one described in Table 4.

## Appendix A.

### Appendix A.1. Mass and Heat Transfer Limitations

#### Appendix A.1.1. External Mass Transfer Limitations

To examine whether the mass transfer between bulk and catalyst pores limited the observed reaction rate, a closer look was taken at the transport phenomena inside the film layer. The transport through the film layer can be described with an external mass transfer coefficient and a driving force.

(A1) $$J=k_{\mathrm{ext}}\left(c_{0}-c_{s}\right)$$

where J is the molar flux through the film layer, $k_{\mathrm{ext}}$ is the external mass transfer coefficient of the film layer, and $c_{0}$ and $c_{s}$ are the concentrations in the bulk and the solid interface, respectively. To determine if this mass transfer step limited the reaction rate, a comparison was made regarding the observed reaction rate. The Mears criterion was used to evaluate this. If the Mears criterion is satisfied, it can safely be said that the effect of the external mass transfer is negligible compared to the kinetically predicted reaction rate. The Mears criterion is satisfied when the ratio of the observed reaction rate and the predicted mass transfer rate is smaller than 0.15. This criterion was based on the limit where the observed reaction was, at most, 5% of the maximum external mass transfer rate [54,55].

(A2) $$C_{M}=\frac{R_{i}^{\mathrm{obs}}r_{p}}{k_{\mathrm{ext}}c_{i}}<0.15$$

where $R_{i}^{\mathrm{obs}}$ and $C_{i}$ are the observed rate of conversion per unit catalyst volume and the bulk concentration of species i. The observed rate of conversion per unit catalyst volume was calculated from the experimentally observed reaction rate using the following relation.

(A3) $$R_{i}^{\mathrm{obs}}=\frac{X_{i}F_{i}^{\mathrm{in}}}{w_{\mathrm{cat}}{\;V}_{R}\left(1-\varepsilon \right)}$$

where $X_{i}$ is the observed conversion of component i, $F_{i}^{\mathrm{in}}$ is the molar feed flowrate of I, and $w_{\mathrm{cat}}$ is the weight fraction of catalyst in the bed. It was assumed that the inert dilutant had the same mass density as the catalyst.

The bed porosity ε was estimated using an empirical correlation by Pushnov et al., which gives the bed porosity for a given particle and reactor diameter. This correlation is given below [56].

(A4) $$\varepsilon =\frac{A}{{\left(\frac{D_{\mathrm{bed}}}{d_{p}}\right)}^{n}}+B$$

For spherical particles, the empirical coefficients A, B and n have values of 1.0, 0.375, and 2.

To determine the external mass transfer coefficient, the Sherwood number was determined. This is often done using an empirical mass transfer correlation described by the Schmidt and Reynolds numbers. From the Sherwood number, the mass transfer coefficient can then be determined. The following correlation for external mass transfer in a fixed bed with spherical particles by Wakao et al. was used [46].

(A5) Sh=2+1.1 Sc13 Re0.6 for 3.0<Re<10,000

When the Sherwood number is known, the external mass transfer coefficient can be calculated with the definition of the Sherwood number, given in Equation (A6). The Sherwood number gives the ratio of total mass transfer rate and diffusional mass transfer rate.

(A6) $$\mathrm{Sh}=\frac{k_{\mathrm{ext}}}{D/d_{p}}$$

where $d_{p}$ is the diameter of the catalyst particle and D is the diffusivity of the gas. Two other dimensionless numbers are required to fulfill Equation (A7), namely, the Schmidt number, giving the ratio of momentum diffusion and the mass diffusion rate, and the Reynolds number, giving the ratio of the inertial and viscous forces [57].

(A7) $$\mathrm{Sc}=\frac{\mu }{\rho D}$$

(A8) Re=ρgusdpμ

where μ is the dynamic viscosity of the gas, $\rho_{g}$ is the mass density of the gas, and $u_{s}$ is the superficial velocity. To estimate the gas diffusivity, the following correlation for the dependency of binary diffusivity on pressure and temperature was used [57]. As a simplification, one general gas diffusivity was used for all gasses (O_2_-N_2_).

(A9) $$D_{\mathrm{AB}}\left(T_{2},p_{2}\right)=D_{\mathrm{AB}}\left(T_{1},p_{1}\right)\frac{p_{1}}{p_{2}}\;{\left(\frac{T_{2}}{T_{1}}\right)}^{1.75}$$

To determine the viscosity in the desired conditions, an empirical correlation using the principle of corresponding states was used. In this correlation, the reduced viscosity is given for any value of the reduced temperature and reduced pressure. The reduced quantity is a dimensionless quantity related to the value of this quantity at the critical point. The graph relating the reduced viscosity, reduced temperature, and reduced pressure, as well as values for the critical properties, can be found in Transport Phenomena by Bird, Stewart, and Lightfoot [58].

The gas density was determined using the ideal gas law and molecular mass.

(A10) $$\rho_{g}=\frac{\mathrm{pM}}{\mathrm{RT}}$$

#### Appendix A.1.2. Internal Mass Transfer Limitations

The mass transfer mechanism can be subdivided in two steps, namely, external mass transfer, which was discussed in the section above regarding internal mass transfer. Internal mass transfer relates to the diffusion of species inside the pores of the catalyst. To examine whether the internal mass transfer limits the observed reaction rate, the Weisz–Prater criterion is often determined. The Weisz–Prater criterion is calculated using the observed reaction rate and internal rate of diffusion. If this criterion is satisfied, pore diffusion can be neglected, since the reaction is much slower [59].

(A11) $$C_{\mathrm{WP}}=\frac{-R_{i}^{\mathrm{obs}}r_{p}^{2}}{D_{\mathrm{eff}}{\;c}_{i}}<1.0$$

The rate of diffusion inside the pores of the catalyst can be described by Fick’s law, however, the type of diffusion determines the actual diffusion coefficient. Diffusion in pores can either be described by molecular diffusion, Knudsen diffusion, or a combination of the two. In the case of molecular diffusion, the stagnant gas in pores is the biggest resistance against molecules traveling through the pore. For Knudsen diffusion, collisions of gas molecules with the walls of the pore determine the diffusion rate. To determine which diffusion coefficient described the diffusion in the pores of the catalyst best, the Knudsen number was determined. The Knudsen number gives the ratio of the mean free path of the gas molecules and the pore diameter. When the Knudsen number is larger than 10, Knudsen diffusion must be considered.

(A12) $$\mathrm{Kn}=\frac{\lambda }{d_{\mathrm{pore}}}>10$$

(A13) $$\lambda =\frac{k_{B}T}{\sqrt{2}{\pi \sigma }^{2}p}$$

where λ is the mean free path of the gas, $d_{p}$ is the catalyst pore diameter, σ is the molecular collision diameter, and $k_{B}$ is the Boltzmann constant. If Knudsen diffusion must be considered, the following expression is used to describe the diffusion coefficient inside the pores where the diffusion mechanism is a combination of molecular and Knudsen diffusion.

(A14) $$D_{\mathrm{Kn}}=\frac{d_{\mathrm{pore}}}{3}\;\sqrt{\frac{8\mathrm{RT}}{\pi M}}$$

(A15) $$\frac{1}{D_{\mathrm{Kn}-M}}=\frac{1}{D_{\mathrm{Kn}}}+\frac{1}{D_{M}}$$

(A16) $$D_{\mathrm{eff}}=\frac{\varepsilon_{p}}{\tau }D_{\mathrm{Kn}-M}$$

where $D_{\mathrm{Kn}-M}$, $D_{\mathrm{Kn}}$, and $D_{M}$ are the combined, Knudsen, and molecular diffusivities, respectively, $\varepsilon_{p}$ is the particle porosity, and τ is the particle tortuosity [60].

An alternative approach used to show the impact of internal mass transport on the reaction rate is the effectiveness factor, which is often described in terms of the Thiele modulus. The Thiele modulus is the square root of the ratio of the reaction rate without any intraparticle limitations and the intraparticle diffusion rate. In Equation (A17), the Thiele modulus $\phi_{1}$ of a first-order reaction is given [57].

(A17) $$\phi_{1}^{2}=\frac{k_{1}r_{p}^{2}}{D_{\mathrm{eff}}}$$

Since accurate reaction rate coefficients of the actual catalytic reaction were not known, an approximation was made. The first-order reaction rate coefficient $k_{1}$ was estimated using experimental data by taking the quotient of the reaction rate and the bulk concentration.

(A18) $$k_{1}=\frac{R_{i}^{\mathrm{obs}}}{c_{i}}$$

The Thiele modulus was used to calculate the effectiveness factor η, which is defined as the ratio of the observed reaction rate and the reaction rate neglecting intraparticle diffusion limitations. Equation (A19) gives the relation of the effectiveness factor and the Thiele modulus of a first-order reaction in a spherical catalyst particle [57].

(A19) $$\eta =\frac{3}{\phi_{1}^{2}}\left(\phi_{1}\coth\phi_{1}-1\right)$$

In this section, two different approaches to characterize the (potential) effect of internal mass transfer on the observed reaction rate are presented. The Weisz–Prater criterion evaluated whether the effect of internal mass transfer was significant or not, while the effectiveness factor and Thiele modulus gave indications regarding the quantification of this effect.

#### Appendix A.1.3. Heat Transfer Limitations

Besides checking potential mass transfer limitations, heat transfer limitations are also potential limitations of the system. Due to the many similarities between the mechanisms of mass and heat transfer, heat transfer is not be discussed as extensively as mass transfer in this work. The Sherwood number for heat transfer directly translates to the Nusselt number, while the Schmidt number for heat transfer translates to the Prandtl number.

The Mears criterion was used to determine whether the external mass transfer limitations were negligible. Besides a criterion for external mass transfer, Mears also describes criteria for both internal and external heat transfer in a fixed bed reactor. The internal and external heat transfer criteria by Mears are shown below [55,61].

(A20) $$\frac{-\left|\Delta H\right|R_{i}^{\mathrm{obs}}r_{p}^{2}}{{\lambda T}_{0}}<0.75\frac{T_{0}R}{E_{a}}$$

(A21) $$\frac{-\left|\Delta H\right|R_{i}^{\mathrm{obs}}r_{p}}{{\mathrm{hT}}_{0}}<0.15\frac{T_{0}R}{E_{a}}$$

where ΔH is the reaction enthalpy, λ is the thermal conductivity of the catalyst particle, h is the external heat transfer coefficient, R is the universal gas constant, $E_{a}$ is the activation energy, and $T_{0}$ is the bulk temperature. Normally, the surface temperature is used for the internal heat transfer criterion, but in this case the bulk temperature was used as an estimation.

The heat transfer coefficient was estimated with a Nusselt correlation using the Prandtl and Reynolds numbers. The used correlation was the same as that used for the external mass transfer coefficient, but Wakao et al. also showed that it was valid for the transport of heat [62].

(A22) $$Nu=2+1.1{\;Pr}^{1/3}{Re}^{0.6}\;\mathrm{for}\;3.0<Re<10,000$$

The Nusselt number Nu represents the ratio of the convective to conductive heat transfer in the gas layers around the particle. The Prandtl number gives the ratio of the diffusion of momentum to the diffusion of heat in the gas layers around the particle.

(A23) $$Nu=\frac{{hd}_{p}}{k}$$

(A24) $$Pr=\frac{Cp\;\mu }{k}$$

where h is the external heat transfer coefficient, k is the thermal conductivity of the gas, and Cp is the specific heat of the gas.
